# Correlation of MRI and arthroscopic findings with clinical outcome in temporomandibular joint disorders: a retrospective cohort study

**DOI:** 10.1186/s13005-021-00305-y

**Published:** 2022-01-07

**Authors:** Kobbe Vervaeke, Pieter-Jan Verhelst, Kaan Orhan, Bodil Lund, Daniel Benchimol, Fréderic Van der Cruyssen, Antoon De Laat, Reinhilde Jacobs, Constantinus Politis

**Affiliations:** 1 Department of Oral and Maxillofacial Surgery, University Hospitals Leuven, Campus Sint-Rafaël, Kapucijnenvoer 33, BE-3000 Leuven, Belgium; 2 OMFS IMPATH Research Group, Department of Imaging and Pathology, Faculty of Medicine, KU Leuven, Leuven, Belgium; 3 Department of Dentomaxillofacial Radiology, Faculty of Dentistry, Ankara University, Ankara, Turkey; 4 Department of Clinical Dentistry, Faculty of Medicine, University of Bergen, Bergen, Norway; 5 Department of Oral and Maxillofacial Surgery, Haukeland University Hospital, Bergen, Norway; 6 Department of Dental Medicine, Karolinska Institutet, Stockholm, Sweden; 7 Department of Oral Health Sciences, KU Leuven, Leuven, Belgium; 8 Department of Dentistry, University Hospitals, Leuven, Belgium

**Keywords:** Arthroscopy, Crumpled disc, Degenerative joint disease, MRI, Temporomandibular disorders, Temporomandibular joint

## Abstract

**Background:**

Arthroscopy is a minimally invasive diagnostic tool and treatment strategy in patients suffering from temporomandibular disorders (TMD) when conservative treatment fails. This study aimed to find specific variables on pre-operative MRI or during arthroscopy that could predict success of arthroscopic lysis and lavage.

**Methods:**

This retrospective analysis compared pre-operative maximum interincisal opening (MIO), pain and main complaint (pain, limited MIO or joint sounds) with results at short-term and medium-term follow-up (ST and MT respectively). Different variables scored on MRI or arthroscopy were used to make a stepwise regression model, subsequently a combined analysis was conducted using variables from both MRI and arthroscopy.

**Results:**

A total of 47 patients (50 joints) met the inclusion criteria. The main complaint improved by 62 and 53% at ST and MT respectively. The absolute or probable absence of a crumpled disc scored on MRI predicted success at ST and MT (*p* = 0.0112 and *p* = 0.0054), and remained significant at MT in the combined analysis (*p* = 0.0078). Arthroscopic findings of degenerative joint disease predicted success at ST (*p* = 0.0178), absolute or probable absence of discal reduction scored during arthroscopy significantly predicted success in the combined analysis at ST (*p* = 0.0474).

**Conclusion:**

To improve selection criteria for patients undergoing an arthroscopic lysis and lavage of the TMJ, future research might focus on variables visualized on MRI. Although more research is needed, disc shape and in particular the absolute or probable absence of a crumpled disc might be used as predictive variable for success.

## Introduction

Temporomandibular disorders (TMD) are a heterogeneous group of conditions involving the temporomandibular joint (TMJ) and associated structures. Patients suffering from TMD most often present with pain, limited mouth opening and joint sounds [1, 2]. The prevalence of TMD is highest in young and middle-aged women (20–50 years), female-to-male ratio ranges from 3:1 to 9:1. Women also seek treatment three times more often than men [2–4]. Approximately 85 to 90% of patients benefit from non-invasive treatment such as reduction of joint loading by education, occlusal splints, NSAIDs or other pharmacological strategies. Generally, non-surgical treatment needs to be continued for at least three to six months before more invasive therapies such as arthrocentesis or arthroscopy are considered [1, 2].

Arthroscopy of the TMJ was first described by Onishi in 1975 [5]. In 1986, Sanders described the arthroscopic lysis and lavage for the treatment of internal derangement with persistent closed lock. The term lysis indicates that adhesions in the superior compartment of the joint are detached. At the same time, intra-articular inflammatory substances are washed out by the lavage [6]. Since then this technique has been used in the management of internal derangements and degenerative joint disease (DJD). Success rates of arthroscopic lysis and lavage in short-term and long-term follow-up studies are on average between 54 and 80% when it comes to pain reduction and increase in mouth opening [7–12].

In therapy-resistant cases, additional imaging of the TMJ is often required. MRI is the gold standard to evaluate the soft tissue components of the TMJ and is effective for the detection of early signs of TMJ dysfunction like joint effusion, changes in disc shape and position, and thickening of the anterior or posterior band [13]. While MRI is also reliable for the detection of internal derangement, especially for anterior disc displacement with reduction (ADDwR), the diagnostic accuracy for evaluating the presence of intra-articular adhesions is rather poor [14–16].Joint effusion seen on MRI has been examined as a potential predictor for successful arthroscopy in the past but was not associated with better postoperative outcome [17].

Previous research focused mainly on clinical parameters for predicting success of arthroscopy.

Psychiatric disorders, concurrent use of benzodiazepines, high self-graded global pain, bilateral muscle tenderness at palpation and small MIO are all correlated with a negative outcome [7, 18, 19]. A recent retrospective study of Muñoz-Guerra et al. also showed no significant correlation between age and postoperative improvement referred to pain or MIO [11].

Because MRI and arthroscopy are commonly done in the refractory TMD patient group, it would be beneficial to identify which diagnostic findings are associated with a higher success rate of the lysis and lavage effect of arthroscopy. In a previous article we already assessed the correlation between MRI and arthroscopic findings was. We found that the agreement between MRI and arthroscopy was poor, which means that when blinded to clinical information MRI and arthroscopic observations can lead to different conclusions. It is therefore important to combine both examinations with clinical information to arrive to a final diagnosis [20]. In this article we focus more on the clinical side. The aim of this study therefore is to identify predictive variables to help physicians predict which patients might benefit the most from an arthroscopy.

## Material and methods

### Study design and patients

All patients who had a diagnostic arthroscopy of the TMJ at the Department of Oral and Maxillofacial Surgery at the University Hospital of Leuven, Belgium, during a five-year period (01-10-2013 until 31-10-2018) were reviewed. A total of 47 patients (50 joints) met the inclusion criteria and were included in this single-centre, retrospective observational study. Ethical approval was given by the Research Ethics Committee UZ/KU Leuven (MP 012487).

Patients of all ages and genders were included if they met following criteria: 1) An MRI of the TMJ was taken no more than six months before the arthroscopy and MRI imaging was available through exportable DICOM data, 2) A video recording of the arthroscopy was available, 3) Preoperative clinical examination parameters were available. Patients were excluded if they did not meet the criteria listed above or if they: 1) Did not have a follow-up appointment in the six weeks after arthroscopy, 2) If a concomitant TMJ surgery was done during arthroscopy.

### Clinical variables

Patient files were searched for clinical variables prior to surgery, at the short-term follow-up (ST) less than six weeks after surgery and at the first medium-term follow-up (MT) more than six weeks after surgery. Preoperative maximum inter-incisal opening (MIO) (mm), presence of pain in the TMJ (Yes/No) and presence of TMJ sounds (Yes/No) were extracted from the patient files. The symptom that interfered most with their daily living activities was categorized as the patient’s main complaint. MIO and the presence of pain and sounds were scored again at both short-term and medium-term follow-up. A limited mouth opening, defined as MIO of 35 mm or less (yes/no) was registered as well as a general improvement in pain (yes/no) and improvement in main complaint (yes/no). Each joint received one or more diagnostic labels based upon clinical findings prior to surgery; Anterior Disc Displacement without Reduction (ADDwoR), Anterior Disc Displacement with Reduction (ADDwR), Degenerative Joint Disease (DJD), Inflammatory Arthritis (IA), Fibrous Ankylosis and ‘Others’.

### MRI and arthroscopy

A scoring system was developed to evaluate the different structures of the TMJ on MRI and arthroscopic images in a reproducible and standardized manner (Table 1). All MRI and arthroscopic images were pseudonymised and evaluated by one oral and maxillofacial radiologist and surgeon respectively. A tentative diagnosis was made based upon these findings. These variables were then correlated with clinical parameters to check for findings that could predict success of arthroscopy.

**Table 1:** Scoring system for MRI and arthroscopy

	MRI	Arthroscopy
Discal displacement	Likert	Likert
Anterior	Likert	Likert
• Posterior	Likert	Likert
• Partial	Likert	Likert
• Reduction	Likert	Likert
Discal deformation	Likert	Likert
• Crumpled disc	Likert	Likert
• Rounded disc	Likert	Likert
• Flat disc	Likert	Likert
• Disc perforations	Likert	Likert
Adhesions	Likert	Likert
Joint effusion	Likert	–
Lateral pterygoid muscle anomalies	Likert	–
Pterygoid shadow anomaly	–	Likert
Retrodiscal deformation	Likert	Likert
Glenoid fossa deformation	Likert	Likert
• Chondromalacia grade	–	Grading
Articular eminence deformation	Likert	Likert
• Chondromalacia grade	–	Grading
Condylar deformation	Likert	–
Overall synovitis	–	Likert
Haemorrhagic infiltrations		
• Anterior band of the disc	–	Grading
• Posterior band of the disc	–	Grading
• Retrodiscal synovium	–	Grading
• Anterior recess	–	Grading
• Fossa	–	Grading
• Articular eminence	–	Grading
• Medial synovium	–	Grading

*Likert scale 1 to 5*:

1. Absolutely not

2. Probably not

3. Uncertain

4. Probably yes

5. Absolutely yes

*Synovitis Grading*

0. Normal, with whitish synovial tissue and wire capillaries

1. Light hypervascularity, with larger capillaries

2. Moderate hypervascularity, with the presence of petechiae

3. Severe hypervascularity, with the presence of broad areas of petechiae/ecchymosis

*Chondromalacia Grading*

0. Normal, completely smooth, smooth white surface with striations on the surface

1. Surface degeneration

2. Intermediate degeneration, with fibrocartilage with bubbles, but without bone exposure

3. Advanced degeneration, with evident bone exposure throughout the joint

If a variable is not assessed on MRI or arthroscopy, it is indicated with ‘-‘

All arthroscopic procedures were conducted by the same experienced maxillofacial surgeon using a 1.9 mm and 30° arthroscope with two 2.7 outer protective cannulas as described by McCain and colleagues [21]. An arthroscopy in our centre is performed in following patients: persistent pain in the TMJ that is arthrogenous of origin AND the pain did not respond to at least three months of conservative treatment AND the pain is of such nature that it interferes with the patient’s daily life. The decision to inject intra-articular corticoids (Diprophos®, betamethasone) was made based upon the perioperative arthroscopic findings of the degree of inflammation of the synovium. Hyaluronic acid was only injected if there was evidence of severe degenerative joint disease.

### Statistical analysis

Data were analysed using IBM SPSS Statistics for Windows, version 26. Fisher’s exact test was used for bivariate data and the Student t-test for mean values. A stepwise regression model (bidirectional elimination) was used for making a model selection. First, a separate model was made for MRI and arthroscopy, subsequently a combined analysis was conducted. A *P*-value of less than 0.05 was set to be significant.

## Results

### Preoperative demographic data

A total of 47 patients (50 joints) met the inclusion criteria (Table 2). Two patients underwent an arthroscopy of both TMJ’s on the same day. One patient presented with similar complaints in the other TMJ after a first arthroscopy. She had a second arthroscopy on the other side 21 months after the first one. All patients had a post-operative appointment within six weeks after surgery with an average of 1.5 weeks (ST) (SD = 0,76), while 36 patients also had a medium-term follow-up appointment, on average 5.4 months after arthroscopy (MT) (SD = 1,74).

**Table 2:** Patients characteristics

		n	%
Patients		47	100%
	Male	5	11%
	Female	42	89%
	Age (years)	41 (mean)	16 (std)
	Time First Consult-Arthro (months)	18.9 (mean)	34,5 (std)
	Time MRI-Arthro (days)	70,82 (mean)	43,97 (std)
Intra-articular injection	Corticosteroids	44	88%
	Hyaluronic acid	4	8%
	None	2	4%
Main complaint	Pain	42	89%
	Limited maximum interincisal opening	4	9%
	Sounds	1	2%
Diagnostic labels	Anterior disc displacement without reduction	20	40%
	Anterior disc displacement with reduction	3	6%
	Degenerative joint disease	23	46%
	Inflammatory arthritis	28	56%
	Fibrous ankylosis	5	10%
	‘Other’	2	4%

Age stands for the age at the time of arthroscopy. Time First Consult-Arthro is the time in months between the first consultation at our department and the diagnostic arthroscopy. Time MRI-Arthro is the time between the MRI scan and the arthroscopy. Limited MIO: a Maximum Interincisal Opening of ≤35 mm

### Surgical outcome

Average MIO did not differ significantly between the pre-operative consultation, the short-term and the medium-term follow-up (Table 3). Overall subjective improvement in main complaint was observed in 62% of patients at the ST and in 53% at the MT. There was no significant difference in improvement in main complaint between men and women (*p* = 0.6348) at the short-term nor at the medium-term follow-up (*p* = 1).

**Table 3:** Surgical outcome

	Pre-operative	Short-term follow-up	Medium-term follow-up
Average MIO (mm)	33.17 mm (SD = 9.97)	33.02 mm (SD = 8.59)	34.75 mm (SD = 7.34)
Limited MIO (%)	66% (31/47)	62% (29/47)	56% (20/36)
Pain (%)	100%(47/47)	60% (28/47)	67% (24/36)
Objective improvement in MIO		32% (15/47)	29% (10/36)
Subjective improvement in MC		62% (29/47)	53% (19/36)
Subjective improvement in pain if MC was pain		62% (29/47)	50% (18/36)

MIO: Maximum Interincisal Opening, Limited MIO: a Maximum Interincisal Opening of ≤35 mm, MC: Main Complaint

### Stepwise regression model

#### Separate analyses

**Fig. 1:**
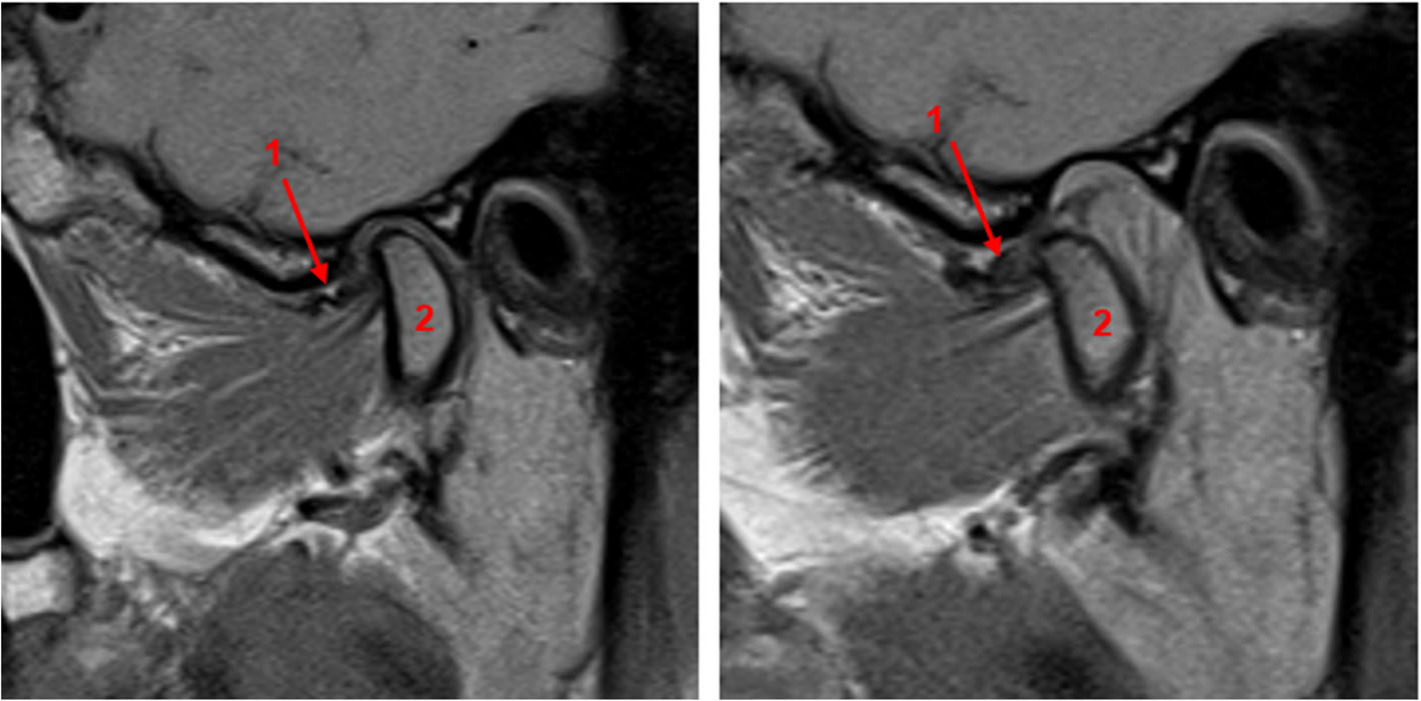
Example of a patient with a crumpled disc [1] visualized on T1-weighted MRI. The left image was taken with a closed mouth, the right image with an open mouth. Also note the anterior disc displacement without reduction. The condyle [2] only showed minor degenerative changes

In the analysis of the MRI, the absolute or probable absence of a crumpled disc (Fig. 1) was a significant predictor for improvement of the main complaint at short-term and medium-term follow-up (*p* = 0.0112 and *p* = 0.0054) (Table 4). In the patient group with pain as main complaint, the absolute absence of any sign of articular eminence deformation was the only variable significantly correlated with improvement of pain in ST and MT (*p* = 0.0052 and *p* = 0.0325). In the separate analysis for variables scored on arthroscopic images, only DJD significantly correlated with ST improvement of the main complaint (*p* = 0.0178).

**Table 4:** Stepwise regression model

		Variable	*P*-Value
MRI	ST improvement in MC in the entire population	Crumpled disc ≤3	0.0112
		Rounded disc ≤1	0.073
		Articular eminence deformation ≤1	0.0848
		Lateral pterygoid muscle anomaly ≤2	0.1763
		Joint effusion ≤2	0.2095
	ST improvement in MC if MC is pain	Condylar Deformation ≤4	**0.0029**
		Lateral pterygoid muscle deformation ≤2	**0.0049**
		Articular eminence deformation ≤1	**0.0052**
		Rounded disc ≤1	**0.0227**
	MT improvement in MC in the entire population	Crumpled disc ≤3	**0.0054**
		DJD	0.1861
		Inflammatory arthritis	0.1992
	MT improvement in MC if MC is pain	Articular eminence deformation ≤1	**0.0325**
Arthroscopy	ST improvement in MC in the entire population	DJD	**0.0178**
		Haemorrhagic infiltration of the medial synovium ≤1	0.0845
		Other	0.068
	ST improvement in MC if MC is pain	Reduction ≤2	0.0744
		Other	0.4226
	MT improvement in MC in the entire population	Displacement of the disc ≤4	0.058
	MT improvement in MC if MC is pain	Articular eminence deformation ≤4	0.2853
MRI + Arthroscopy	ST improvement in MC in the entire population	Reduction (arthroscopy) ≤ 2	**0.0474**
		Other (arthroscopy)	0.3772
	ST improvement in MC if MC is Pain	DJD (arthroscopy)	0.0559
		Other (arthroscopy)	0.1823
		Joint effusion (MRI) ≤ 1	0.1418
	MT improvement in MC in the entire population	Crumpled disc (MRI) ≤ 3	**0.0078**
		DJD (MRI)	0.0581
	MT improvement in MC if MC is Pain	Articular eminence deformation (arthroscopy) ≤ 4	0.9984

MRI: Magnetic resonance imaging, ST: short-term, MT: medium-term, MC: Main Complaint, DJD: Degenerative Joint Disease. *P*-values in bold are significant. 5-point Likert scale (1. absolutely not present; 2. probably not present; 3. uncertain; 4. probably present; 5. absolutely present)

#### Combined analysis

The absolute or probable absence of discal reduction observed on arthroscopic images correlated significantly with improvement of the main complaint (*p* = 0.0474), although in the short-term only. At the medium-term follow-up, the absolute or probable absence of a crumpled disc scored on MRI (*p* = 0.0078), remained a significant predictor for improvement of main complaint. Unfortunately, there was too little data to make any analysis for patients who designated limited mouth opening or joint sounds as their main complaint.

## Discussion

With a subjective improvement of the main complaint in 62% of cases at short-term follow-up and 53% at medium-term follow-up, our success rates are rather low if compared to previous studies [7, 9, 18, 19, 22]. Ulmner and colleagues found in their short-term prospective study that only 11% of patients with DJD, 20% with ADDwoR and 36% with chronic inflammatory arthritis showed no or little improvement after arthroscopy [19]. Haeffs et al. on the other hand only had a successful surgical outcome in 62.3% of patients with TMJ arthralgia or internal derangement [7]. Other reports describe successful outcomes in 67% of patients with arthralgia with or without OA, rheumatic disease or chronic closed lock, in 80% of patients with ADDwoR and 86,7% in patients with internal derangement [9, 18, 22]. Muñoz-Guerra et al. had a success rate of 54.24% in patients with internal derangement [11]. It is hard to have a direct comparison of all these results considering heterogeneity in disease classification, presentation, and follow-up. Although more recently, diagnostic criteria for temporomandibular disorders, known as the DC/TMD, are available they are not universally used in clinical practice [23, 24]. Secondly, the duration of the symptoms before the arthroscopic intervention might play an important role in the success of arthroscopic lysis and lavage [9, 25]. The average time between the first consult and the arthroscopy was 18.9 months. Israel et al. found a negative correlation between duration of the symptoms and success of an arthroscopy in patients with inflammatory or degenerative TMJ diseases [25]. Thirdly, it is important to note that only 36 out of the 47 patients attended a MT appointment which leads to a possible incomplete representation of MT success rates.

Hyaluronic acid was injected during the arthroscopic procedure in four joints where there were signs of severe degenerative joint disease as it might have a positive effect on pain reduction [26]. However, a recent randomized controlled trial published in 2021 with 51 patients (Wilkes stage-III and stage-IV) observed no beneficial effect of hyaluronic acid on pain reduction compared to arthroscopy alone [27]. These new insights were not available at the time the arthroscopies were performed but are important to take into account when performing arthroscopies in the future. There were too few patients who received hyaluronic acid in this study to draw any conclusions of its effect on reducing pain.

In a previous study we showed the poor correlation between arthroscopic and MRI findings in patients with TMD. This means that when blinded to clinical information MRI and arthroscopic observations can lead to different conclusions. There was only a fair agreement reached for the reduction capacity of the disc and disc perforation [20]. In this study our main aim was to find identify different variables observed during MRI and/or arthroscopy that are significantly correlated with success of the arthroscopic lysis and lavage. On MRI, an irregular, crumpled or rounded disc is mostly seen in later stages of internal derangement. In the early stages, the disc retains its normal shape [28]. The fact that the absolute or probable absence of a crumpled disc and the absolute absence of a rounded disc deformation, i.e. absence of clear signs of advanced internal derangement, correlated with better outcomes suggests that early intervention in internal derangement might be beneficial for patients; although care should be taken not to overtreat patients. Some authors also believe that increased thickness of the attachment of the lateral pterygoid muscle can be seen as an indirect sign of progression in TMJ dysfunction and that flattening of the articular eminence can be interpreted as a secondary result of internal derangement [13, 28, 29]. The absolute and probable absence of lateral pterygoid muscle deformations and absolute absence of articular eminence deformations correlated significantly with improvement in pain at the ST, again suggesting the positive effect of the lysis and lavage before signs of the advanced disease become visible on MRI. Other studies support these findings that arthroscopic interventions might be more beneficial in early stages of TMD [9, 25, 30]. For example, in a recent network meta-analysis comparing different treatments for arthrogenous TMDs, Al-Moraissi et al. reported that minimally invasive procedures were more effective than conservative treatments for reducing pain and increasing MIO in patients with internal derangement [30]. .Generally, non-invasive treatment strategies should be tried for at least 3–6 months prior to more invasive treatment modalities [2].

By combining the variables scored on MRI and during diagnostic arthroscopy, we attempted to make a combined prediction model. The absolute or probable absence of discal reduction evaluated on arthroscopic video was the only new significant variable in predicting improvement in main complaint in the short-term follow-up. Absence of discal reduction is mostly seen in patients suffering from ADDwoR. Patients with ADDwoR also tend to respond well on arthroscopy in other studies with success rates of up to 80% [19]. Part of the aetiology of ADDwoR is the presence of adhesions in the upper joint compartment. These adhesions are removed during a lysis and lavage procedure which can explain the success of arthroscopy in ADDwoR [9, 19]. The presence of adhesions during arthroscopy in patients suffering from internal derangement is also linked with a favourable outcome [11].

Our study had some limitations. The retrospective design increased the risk of bias and inaccurate data when reviewing patient records for the extraction of clinical parameters. Furthermore, because all arthroscopic videos were reviewed retrospectively, not being able to manipulate the scope during the assessment made the interpretation of the videos more difficult. In addition, while a stepwise regression model is a valuable tool for approaching considerable numbers of potential variables, it also has some drawbacks. If for example two variables are correlated strongly and both have a good relationship with the outcome, only one of those two variables is included in the model. This has the consequence that included variables can represent a group of variables that correlate strongly between themselves. Besides, a model selection is only successful for those patients where all variables are measured. This explains why results of the combined analysis differ from the separate analyses. Finally, a study population of 50 joints poses a statistical challenge and care should be taken to transfer these results to the general population.

## Conclusion

In conclusion, patients presenting with TMD, should receive conservative treatment including reduction of joint loading by education, occlusal splints, NSAIDs or other pharmacological strategies for at least 3 to 6 months. When no or almost no improvement is observed, an MRI scan can be performed to further evaluate the underlying cause of the symptoms. MRI might also have a great potential in the future in predicting which patients might benefit the most from arthroscopic lysis and lavage. Disc shape and in particular the absolute or probable absence of a crumpled disc might be used as a predictive variable in patients suffering from TMD. The absence of eminence deformation on MRI also predicted better outcome in the medium-term follow-up. Perioperative findings such as DJD or absolute or probable absence of disc reduction might predict initial improvement of the main complaint. Future studies are indicated to further evaluate what clinical or radiographic variables will improve selection criteria for patients undergoing an arthroscopy of the TMJ.

## Data Availability

The datasets used and/or analyzed during the current study are available from the corresponding author on reasonable request.

## Abbreviations

ADDwoR: Anterior disc displacement without reduction
ADDwR: Anterior disc displacement with reduction
DJD: Degenerative joint disease
IA: Inflammatory arthritis
MIO: Maximum interincisal opening
MRI: Magnetic resonance imaging
MT: Medium-term follow-up
ST: Short-term follow-up
TMD: Temporomandibular disorder
TMJ: Temporomandibular joint
